# The New York Registry Bill

**Published:** 1896-06

**Authors:** 


### THE NEW YORK REGISTRY BILL.

The bill passed by the New York Legislature on May 22, 1896, provides the privilege of registration for those graduates who had so failed to do during the time allowed for the same, and they may now register through unanimous recommendation of the State Board of Veterinary Medical Examiners to the Board of Regents. But they will not again be thus favored, save through the regular examination; and all those who have failed to register are now enabled so to do, but will receive no further consideration at the hands of the veterinary organizations in the Empire State. Secretary Kelly is anxious that a prompt compliance be made, so that time and money may be spared in seeking needless legislation in the future.

